# Prediction of Breast Radiation Absorbed Dose Chest CT Examinations Using Machine Learning Techniques

**DOI:** 10.3390/tomography11120142

**Published:** 2025-12-16

**Authors:** Sevgi Ünal, Remzi Gürfidan, Merve Gürsoy Bulut, Mustafa Fazıl Gelal

**Affiliations:** 1 Department of Radiology, Ataturk Training and Research Hospital, Izmir Katip Celebi University, Izmir 35150, Türkiye; 2 Isparta Vocational School of Information Technologies, Database, Network Design and Management, Isparta University of Applied Science, Isparta 32100, Türkiye; remzigurfidan@isparta.edu.tr

**Keywords:** chest computed tomography, total DLP, breast-specific radiation dose, machine learning

## Abstract

**Background/Objectives**: The breast is a highly radiosensitive organ that is directly exposed to ionizing radiation during chest computed tomography (CT) examinations. Excessive radiation exposure increases the risk of radiation-induced malignancies, highlighting the importance of accurate and patient-specific dose estimation. This study aims to estimate the effective radiation dose absorbed by the breast during chest CT examinations using a machine learning (ML)-based personalized prediction approach. **Methods**: In this retrospective study, a total of 653 female patients who underwent both mammography and chest CT between 2020 and 2024 were included. A structured database was created incorporating demographic and anatomical parameters, including body weight, height, body mass index (BMI), and breast thickness (mm) obtained from mammography, along with dose length product (DLP) values from chest CT scans. Five regression-based ML algorithms—CatBoost, Gradient Boosting, Extra Trees, AdaBoost, and Random Forest—were implemented to predict breast radiation dose. Model performance was evaluated using Mean Squared Error (MSE), Mean Absolute Error (MAE), Mean Absolute Percentage Error (MAPE), and the Coefficient of Determination (R^2^). **Results**: Among the evaluated models, the CatBoost algorithm optimized with Particle Swarm Optimization (CatBoostPSO) achieved the best overall predictive performance, yielding the lowest MSE (0.3795), MAE (0.3846), and MAPE (4.37%), along with the highest R^2^ value (0.9875). CatBoost and Gradient Boosting models demonstrated predictions most closely aligned with ground truth values, indicating that ensemble-based and dynamically optimized models are particularly effective for breast dose estimation. **Conclusions**: The proposed machine learning framework enables rapid, accurate, and clinically applicable estimation of breast radiation dose during chest CT examinations. This patient-specific approach has strong potential to support personalized radiation dose monitoring and optimization strategies, contributing to improved radiation safety in clinical practice.

## Simple Summary

Breast tissue is highly sensitive to radiation during chest computed tomography scans, and estimating the dose it receives is important for patient safety. This study used patient characteristics such as weight, height, breast thickness, and measured scan dose to predict the breast-absorbed radiation level with machine learning. The model provided highly accurate estimates, offering a fast and practical way to support personalized dose monitoring. These findings may help radiologists optimize imaging protocols, reduce unnecessary radiation exposure, and guide future research on organ-specific dose prediction.

## 1. Introduction

Chest computed tomography (CT) is a cross-sectional imaging modality that utilizes ionizing radiation and is widely employed for the evaluation of pulmonary and mediastinal pathologies [1,2]. However, repeated CT examinations can pose a potential cancer risk for several radiosensitive organs—most notably breast tissue—due to cumulative exposure to ionizing radiation. Although the breast is not the primary target during chest CT, it can still receive a considerable amount of scattered radiation, which may significantly elevate the risk of radiation-induced malignancies in patients undergoing multiple scans [3,4]. The absorbed radiation dose to the breast during a chest CT examination has been reported to be approximately 100 times higher than that of a standard screening mammogram [5].

In CT imaging, the radiation dose is commonly quantified using parameters such as the dose length product (DLP) and the volumetric CT dose index (CTDI<sub>vol</sub>). These indices serve as standardized indicators of patient exposure but do not directly represent organ-specific absorbed doses [6]. To better assess radiation exposure, computed dosimetry software is routinely used to estimate organ and effective doses in CT examinations. Nevertheless, studies have demonstrated substantial variability among different dosimetry software tools, particularly when applied to adult populations [7]. Accurate organ dose determination is essential for evaluating the biological risks associated with ionizing radiation. The absorbed dose, expressed in milligray (mGy), represents the amount of energy deposited per unit mass of tissue by radiation. Conventional dose calculation techniques, however, often neglect individual anatomical variability and physiological differences among patients, thereby limiting their precision. Organ-specific dose estimations that incorporate patient-specific parameters yield the most accurate results but remain impractical for routine clinical use due to their computational and procedural complexity. Consequently, there is a growing demand for practical, individualized dose estimation approaches that balance accuracy and clinical feasibility.

Two commonly employed methods for estimating organ-specific radiation doses are size-specific dose estimation (SSDE) and Monte Carlo (MC) simulation. Although SSDE provides a more personalized measure than traditional indices, it fails to account for individual variations in tissue composition and organ morphology [8,9,10]. Moreover, SSDE relies on standard water phantoms for measurement [11], which limits its anatomical realism. In contrast, MC simulation remains the most accurate technique for organ dose assessment, as it models radiation transport based on detailed reference phantoms that consider parameters such as age, sex, and body size. MC-based approaches can compute personalized organ doses for segmented regions of interest (ROI) with high precision; however, they are computationally intensive and unsuitable for clinical workflows requiring rapid analysis [12,13]. To date, only a limited number of studies have focused specifically on breast dose estimation during chest CT, and those that exist primarily rely on MC simulation methods [14].

Recent advances in artificial intelligence have introduced machine learning (ML) as a powerful alternative for patient-specific dose estimation. ML algorithms can simultaneously analyze multiple clinical and imaging parameters—such as patient age, body mass index (BMI), tube current (mA), tube voltage (kV), and DLP—to predict radiation dose with high accuracy [15]. Studies have shown that ML-based dose estimation can outperform conventional MC simulation in certain applications, demonstrating improved efficiency and comparable or superior accuracy [16]. These findings suggest that ML-based systems hold significant promises for optimizing organ-specific radiation dose management in thoracic CT protocols.

Accordingly, the present study aims to estimate the radiation done by breast tissue during chest CT examinations using advanced machine learning techniques. Additionally, the study investigates the influence of key clinical and anatomical parameters on dose estimation, providing insights into the development of a fast, reliable, and patient-specific breast dose prediction framework.

## 2. Materials and Methods

A total of 653 female patients who underwent mammography between 2020 and 2024 and had a chest CT scan in our database within the last 6 months were included in the study. A structured database was created containing the patients’ weight, height, BMI values, breast thickness (mm) measured on mammography to determine breast density, and dose length product (DLP) values from the patients’ chest CT scans. The performance of the five-regression machine learning (ML) models used—CatBoost, Gradient Boosting, Extra Trees, AdaBoost, and Random Forest—was evaluated in detail. Evaluation metrics such as Mean Squared Error (MSE), Mean Absolute Error (MAE), Mean Absolute Percentage Error (MAPE), and Coefficient of Determination (R^2^) were used to provide a comprehensive overview of the models’ predictive ability and generalization performance. The parameters of each model were optimized using the PSO method and determined automatically.

The dataset was randomly divided into training (80%) and test (20%) sets using the train_test_split function with a fixed random state (random_state = 42). Although stratified splitting on the target dose (IR) was not used, we verified that the dose distribution (mean and range) was comparable between the training and test sets to minimize potential bias.

### Purpose of Mathematical Model and Calculation Methods

The main purpose of this model is to estimate personalized IR (Internal Radiation), which considers patient-specific physical characteristics, in addition to the total DLP (Dose-Length Product) value measured in computed tomography (CT) applications. The model is intended to predict the amount of radiation retained in the breast (absorbed dose), which is difficult to obtain by direct physical measurement. The model structure and general formula are calculated by Equation (1).

(1) $$IR=k*DLP*F_{1}*\;F_{2}*F_{3}$$

- IR: Radiation dose retained in the breast (expressed in mGy or mSv)
- DLP: Total Dose-Length Product, directly measured dose (mGy-cm)
- k: Constant multiplier—an empirical coefficient that depends on the units and system calibration
- BMI: A normalized conversion function reflecting an individual’s tissue density and fat to muscle ratio
- Breast Thickness: Factor affecting the absorption rate of radiation in breast tissue
- Age: Age-related radiobiological effect coefficient

DLP is a measure of the total dose delivered by the radiological device. However, it does not represent individual radiation absorption. Therefore, it must be weighed by individual biophysical factors. This weighting is calculated by Equation (2).

(2) $$BMI=\frac{Weight\;(kg)}{Height(m^{2})}$$

Body mass index (BMI) represents the body density of a person. It is effective in determining how much radiation will penetrate the tissue. The process is carried out with the normalized value of this variable. The normalization process is calculated by Equation (3).

(3) $$F_{1}\left(BMI\right)=\frac{BMI-{BMI}_{min}}{{BMI}_{max}-{BMI}_{min}}$$

The thickness of the breast tissue (CT) is a key variable affecting local dosimetric absorption, especially in thorax CT. The process is carried out with the normalized value of this variable. The normalization process is calculated by Equation (4).

(4) $$F_{2}\left(BT\right)=\frac{BT-{BT}_{min}}{{BT}_{max}-{BT}_{min}}$$

Biological sensitivity to radiation may change with increasing age. The process is carried out with the normalized value of this variable. The normalization process is calculated by Equation (5).

(5) $$F_{3}\left(A\right)=\frac{Age-{Age}_{min}}{{Age}_{max}-{Age}_{min}}$$

The multiplier k in the model is derived from experimental data and is in line with Monte Carlo simulation systems such as NRPB, ICRP or MCNP. The BMI multiplier weight is calculated as 40% as it has a significant effect on the overall tissue absorption. Breast thickness is weighted as 30% and age criterion as 30%. Unlike the Monte Carlo method, this method simulates the distribution of radiation in the body by following thousands of random particle paths. This complex physical simulation is approximated to reality with empirical, weighted and normalized coefficients. Thus, realistic and meaningful personalized dose estimation is obtained.

The weighting coefficients assigned to the normalized BMI, breast thickness and age terms in Equation (2) (0.40, 0.30 and 0.30, respectively) were derived from preliminary correlation and multiple regression analyses in our patient cohort and then refined under the constraint that their sum equals 1, so as to best match IR values calibrated against Monte Carlo–based breast-dose estimates reported in the literature. This choice reflects the empirically observed predominance of body habitus (BMI) over breast thickness and age in determining breast dose.

For each patient, the target variable, actual internal radiation (IR, mGy), was calculated using the DLP measured and patient-specific characteristics via the mathematical model described in Section Purpose of Mathematical Model and Calculation Methods (Equations (1)–(5)). In this model, DLP is multiplied by a body size correction term (“BODY”) composed of normalized BMI, breast thickness, and age, weighted by 0.40, 0.30, and 0.30, respectively, and scaled by an empirical coefficient k calibrated against reference data. IR values obtained independently of machine learning algorithms were accepted as True Values (ground truth) for model training and evaluation.

## 3. Results

Among 653 patients who underwent chest CT, the highest DLP value was 914.5, and the lowest DLP value was 68.81. The breast-specific average radiation dose was calculated using the CatBoostPSO model was 9.76 mGy (1.08–52.7), the average weight was 73.1 kg (46–133), the average height was 1.59 m (1.47–1.77), and the average age was 58.6 (33–88).

Among the five regression machine learning models used, CatBoostPSO demonstrated superior performance across all metrics. It achieved the lowest MSE (0.3795), MAE (0.3846), and MAPE (4.37%) values, while also achieving the highest R^2^ value of 0.9875. The CatBoost and Gradient Boost models stand out as algorithms that produce the closest predictions to real data. These two models showed sensitivity to high-frequency changes and were able to closely follow the actual curves even in extreme values.

### 3.1. Machine Learning Models

In this study, we deliberately focused on five regression algorithms—CatBoost, Gradient Boosting, Extra Trees, AdaBoost, and Random Forest—because tree-based ensemble methods are well-suited for low-dimensional, structured clinical data and can model non-linear relationships and feature interactions effectively with a moderate sample size. Given that our predictors are tabular (weight, height, BMI, breast thickness, age, and DLP) and the cohort size is 653 patients, we did not include deep learning architectures, which typically require larger datasets and image-based inputs to provide a clear performance advantage and are less interpretable in routine clinical practice.

In this case, the performance of five regression machine learning models—CatBoost, Gradient Boosting, Extra Trees, AdaBoost, and Random Forest—that are optimized using the Particle Swarm Optimization (PSO) algorithm is examined in detail. The evaluation metrics considered are Mean Squared Error (MSE), Mean Absolute Error (MAE), Mean Absolute Percentage Error (MAPE), and Coefficient of Determination (R^2^), which collectively provide an overall impression of the predictive capability and generalization performance of the models. The parameters of each model were optimized via PSO, enabling an unbiased and automated search for the most suitable combinations of parameters with which to reduce error on the test data. The results of the experiments are presented in Table 1. Each model’s performance is discussed in detail as follows.

**Figure 1:**
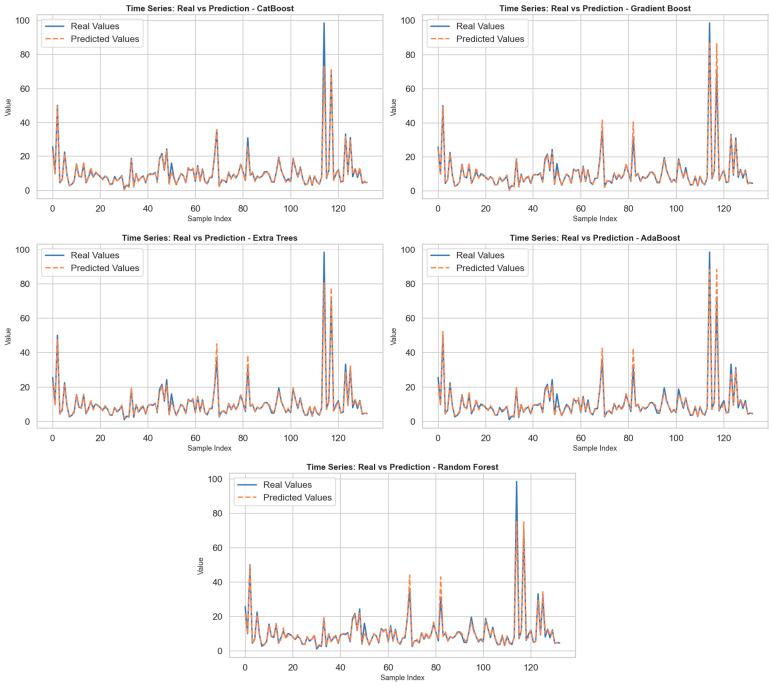
All models, Real and Prediction graph via time series.

CatBoostPSO demonstrated superior performance across all metrics, with the lowest MSE (0.3795), MAE (0.3846), and MAPE (4.37%), while also achieving the highest R^2^ value of 0.9875. The results show that not only is the magnitude of error small, but the explanatory power of the model is also high. This can be ascribed to CatBoost’s ability to handle complex feature interactions as well as its regularization technique that avoids overfitting. The best combination shows that the tree depth was relatively shallow(4) with a moderate value of the learning rate (0.1787), with a compromise between the complexity of the model and its accuracy. GradientBoostPSO also gave promising results with the mean squared error (MSE) value of 1.1458 and the value of R-squared (R^2^) as 0.9622, outperforming conventional ensemble models like Random Forest and AdaBoost. The value of tree depth was biased towards being stable with low overfitting, along with a high number of estimators (200) that helped retain the accuracy. ExtraTreePSO followed closely, maintaining an R^2^ value of 0.9504. However, both MSE and MAE values were moderately higher than those of CatBoost and Gradient Boosting, thus indicating a slightly less robust fit. The relatively deep trees (depth = 12) in the best configuration may have led to increased variance. AdaBoostPSO and RandomForestPSO showed comparably weaker performance, with MSE values above 2.4. Although all these models still achieved R^2^ scores higher than 0.91, the higher MAE and MAPE values imply lower precision in pointwise predictions. Notably, the used base estimator in AdaBoost adopted a tuned tree depth of 7, indicating attempts to capture more non-linear relationships; however, the performance lagged behind boosting methods such as CatBoost. In general, the experiments confirm that gradient-based ensemble methods, in particular CatBoost, significantly outperform bagging-based methods such as Random Forest in terms of both predictive accuracy and generalization capability when optimized using swarm intelligence. By incorporating PSO, the search process for the best hyperparameters was carried out in a more efficient, unbiased manner, thus proving the value of metaheuristic optimization in machine learning algorithms. In the figures produced for every model, the actual value as well as the predictions were shown through the use of the sample number, as shown in Figure 1. By closely examining the figures, the sensitivity of the models to changes over time was determined. Overall, it was found that every model was well able to predict low, medium, and high values, especially well in terms of predicting changes that happened within specific periods. But for those points that experienced sudden jumps in terms of high values, some models were not so good in predicting such extreme values, thus increasing their prediction errors. Specifically, the CatBoost model, along with the Gradient Boost model, can be seen as the algorithms that generated predictions closest to the real value. These two models demonstrated sensitivity to high-frequency changes and were able to closely follow the actual curves even at extreme values. In contrast, while the Extra Trees and AdaBoost models effectively tracked general trends, prediction deviations became more pronounced, especially at peak values. The Random Forest model, on the other hand, produced a relatively smooth prediction curve and diverged from the actual data in some time periods. This is due to the model’s overly smoothing effect. In conclusion, the findings reveal that CatBoost and Gradient Boost models are more successful in problems involving dynamic structures such as time series prediction.

**Figure 2:**
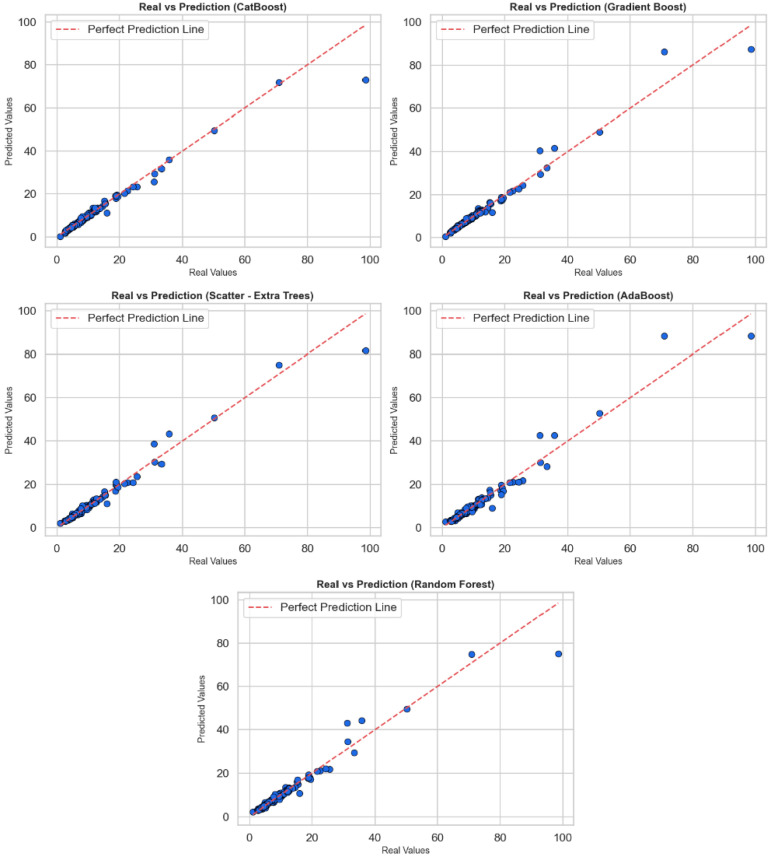
All models, Real and Prediction graph via scatter.

Figure 2 contains scatter plots reflecting the relationship between actual values and predicted values to evaluate the regression performance of CatBoost, Gradient Boost, Extra Trees, AdaBoost, and Random Forest algorithms. In each sub-graph, the horizontal axis shows the actual values, while the vertical axis shows the values predicted by the model; a red dotted line has been added as the perfect prediction line (y = x). The proximity to this line is a visual indicator of the model’s prediction accuracy. When the graphs are examined as a whole, all models make their predictions very close to the actual values in most cases. Most of the points are clustered close to the red reference line, indicating that the models have learned the general trends correctly. However, in each model, deviations from the reference line are noticeable, especially at high-value outliers. These deviations indicate that the models perform relatively poorly in predicting extreme values and that systematic errors may occur in some cases. In particular, the CatBoost and Gradient Boost models are positioned closer to the red line across a wide range of values, indicating that they provide more stable and generalizable predictions. Extra Trees and Random Forest models also perform similarly well, but prediction deviations are observed in a few high-value examples. AdaBoost, on the other hand, provides successful predictions in the low value range but shows more deviation at medium and high levels. In conclusion, these graphs show that models are evaluated not only based on statistical metrics but also in terms of visual consistency. CatBoost and Gradient Boost algorithms stand out in regression accuracy, but all models require careful analysis and potential model improvements at high values.

**Figure 3:**
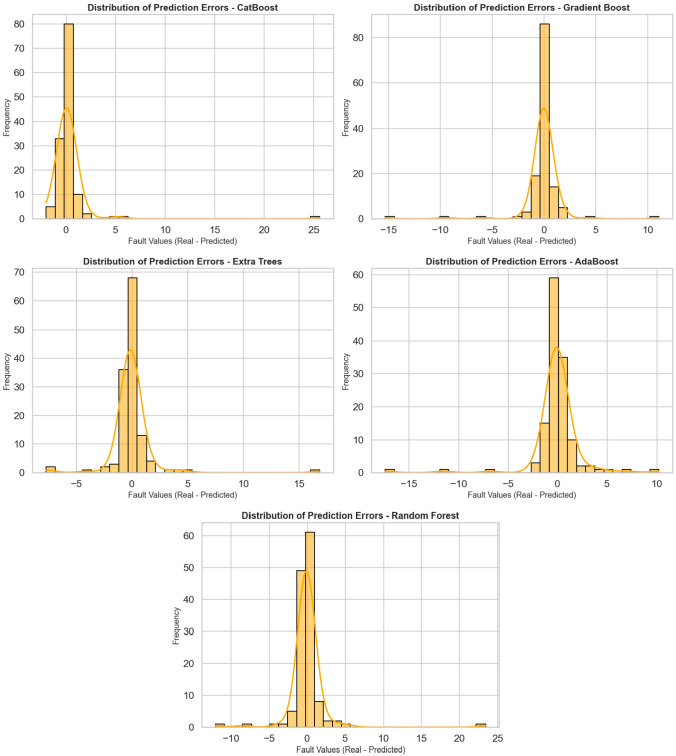
All models’ fault distribution graph.

Figure 3 shows the distribution of prediction errors for five different regression algorithms (CatBoost, Gradient Boost, Extra Trees, AdaBoost, and Random Forest), visualized using histograms and density curves. The horizontal axis represents prediction errors (actual value—predicted value), while the vertical axis represents the frequency of these errors. These graphs are very valuable for analyzing the deviation behavior of models and seeing to what extent predictions are prone to error. When the graphs are examined in general, it can be observed that prediction errors in all models are concentrated close to zero, i.e., within the correct prediction range. In particular, the error distribution in the CatBoost, Gradient Boost, and Extra Trees models is quite symmetrical, with a dense accumulation around the center (around 0). This implies that such models forecast without systematic bias and tend to perform with small errors. The CatBoost model was highly accurate with most errors between −2 and +2. Both the Gradient Boost and Extra Trees models also have a concentrated distribution of errors, but more infrequent but meaningful deviations are recorded at extreme points. The error distribution in the AdaBoost model is more spread out, and there is greater variation between +5 and −5. The Random Forest model primarily follows the central distribution but produces prediction errors greater than 10 for certain outliers. Overall, these distribution charts are crucial to study not only the average performance of the models, but also their reliability and their capacity to respond in unexpected situations. CatBoost and Gradient Boost models demonstrate a stable prediction performance with tight ranges and central density for the distribution of error, whereas for AdaBoost and Random Forest models’ performances, more cautious analyses and deeper optimization are needed due to the deviations of extreme values.

**Figure 4:**
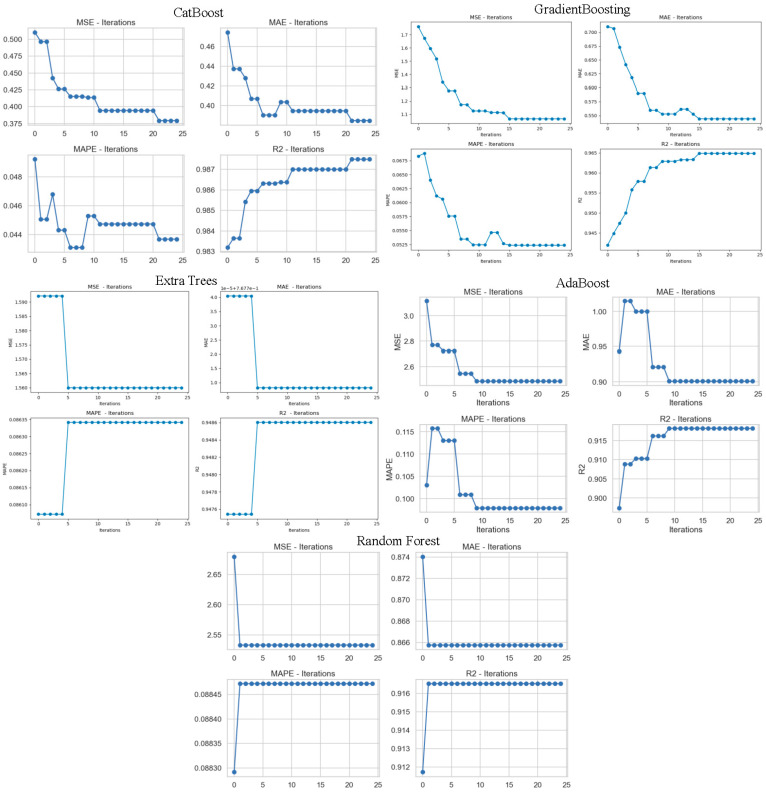
All models’ metric values graph.

Figure 4 juxtaposes how four different regression success metrics (MSE, MAE, MAPE, and R^2^) of the CatBoost, Gradient Boosting, Extra Trees, AdaBoost, and Random Forest models change with model iterations (number of trees or number of iterations in the learning process). These graphs were used to monitor the learning curve and convergence pattern of the models. Each graph provides comprehensive information about stability and performance overall by illustrating the way the model’s performance was enhanced during training. The CatBoost model showed a steady decrease in MSE, MAE, and MAPE values as iterations progressed, indicating that the model learned more with each step. The increase observed in the R^2^ value illustrates that the explanatory power and precision of the model were enhanced throughout. Similarly, for the Gradient Boosting algorithm, improvements in metrics and a steady increase in the R^2^ score were observed, indicating that the model has acquired good generalization power. For the AdaBoost algorithm, there was a sudden drop in error metrics, such as MSE and MAE, especially in the first few iterations, after which the metric values stabilized. This indicates that AdaBoost learns quickly in the first few iterations but then becomes slower. The R^2^ plot also confirms this, plateauing after a while. The Random Forest and Extra Trees models, however, showed negligible variation in the iterations. The reason why the measurements were nearly fixed in these two models is that the algorithms were trained using a certain number of trees, and these structures were not recalculated very often. Therefore, it can be said that these models are efficient in strong initial performance with their ensemble model rather than an iterative improvement mechanism. Overall, CatBoost and Gradient Boosting models are not only good at high performance but also at stable and continuous improvement in learning processes. AdaBoost is efficient with its fast-learning capability, while Extra Trees and Random Forest models are efficient in consistent but solid performance.

In Figure 2 and Figure 3, all models exhibit low and centrally concentrated errors for the majority of low–to–medium breast dose values, whereas larger and more variable residuals are observed for a small number of highest-dose outliers, particularly in the AdaBoost and Random Forest models. This behavior likely reflects the limited number of observations in the extreme upper tail and the smoothing effect of bagging-based ensembles, which prioritize fit in the densely populated central region. CatBoost and Gradient Boost maintain tighter error distributions across almost the entire range, although a slight increase in error is also seen for the rare highest-dose cases. Future work will focus on collecting more high-dose examples and exploring tail-sensitive training strategies to further improve performance for extreme values.

**Figure 5:**
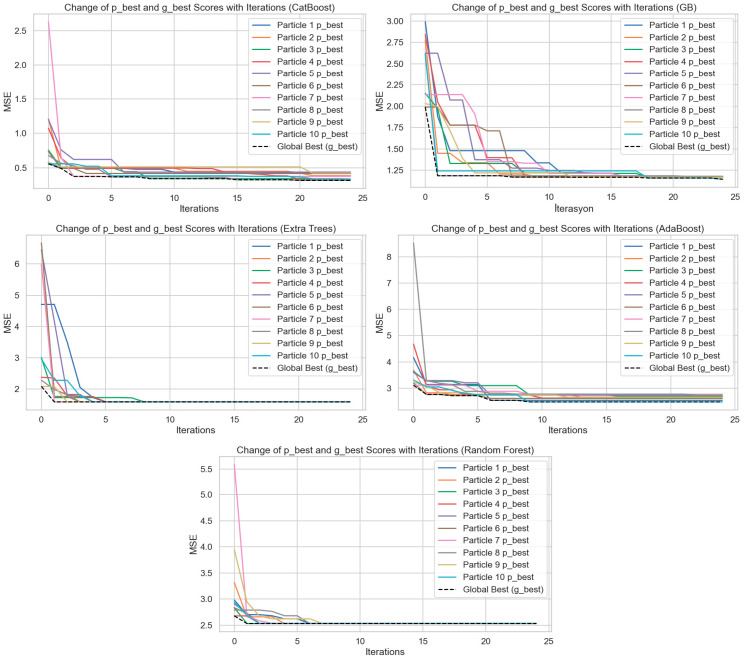
Particle motion graphs according to algorithms.

Figure 5 shows the hyperparameter optimization processes performed by applying the Particle Swarm Optimization (PSO) algorithm to the CatBoost, Gradient Boost (GB), Extra Trees, AdaBoost, and Random Forest models. The plots show how the best score (p_best) of 10 particles and the global best score (g_best) change with the iterations. The *Y*-axis represents Mean Squared Error (MSE), and the *X*-axis represents the iterations. When the graphs are considered collectively, it can be observed that with an increase in iterations in each model, both the particle best scores and the global best scores individually decrease substantially. This condition reflects that the PSO algorithm can optimize the hyperparameter space effectively and obtain lower errors at every iteration. The CatBoost model is observed to have declining MSE values at a fast and consistent rate. A better rate was observed in the first 5 iterations, beyond which both particle differences lowered, and the global best score became constant. The same pattern was observed for the Gradient Boosting algorithm, although particle differences were greater at the initial steps. Scores converged towards each other within approximately 10 iterations. The Random Forest and Extra Trees models both exhibited the same trend of an accelerating convergence pattern, steep drops in MSE values from the first 3–5 iterations. This indicates that the two models had both learned through hyperparameter tuning in the early stages. However, the subsequent stabilization of errors shows that the models reached a limited learning capacity and that further iterations did not provide marginal gains. In the AdaBoost model, the initial MSE values are higher, and there is greater variance among the particles. However, as iterations progress, errors decrease significantly, indicating that AdaBoost can be significantly improved through optimization. Overall, these graphs demonstrate that the PSO algorithm is a powerful tool for minimizing model error by providing effective optimization in the hyperparameter settings of regression models. Additionally, when comparing models, CatBoost and Gradient Boosting algorithms stand out in the optimization process by achieving lower and more stable MSE values. This study visually highlights the critical impact of hyperparameter optimization on model performance.

In this study, the outputs of the mathematical model developed for the prediction of personalized breast radiation dose are evaluated comparatively with different machine learning algorithms. Five different regression algorithms (CatBoost, Gradient Boosting, Extra Trees, AdaBoost and Random Forest) were trained with PSO-based hyperparameter optimization and the generalizability and error levels of the model were analyzed in detail. The results show that the CatBoost algorithm is superior in all performance metrics (MSE, MAE, MAPE, R^2^) and successfully follows time series trends. The Gradient Boosting model performed close to CatBoost and proved to be a strong alternative. The error scatter plots confirm that these two models are free of systematic bias and operate with low variance. The PSO process significantly improved the accuracy of the models and clearly demonstrated the contribution of optimization to model performance. Models such as AdaBoost and Random Forest produced less consistent results, with higher error rates, especially at extreme values. Therefore, in areas that require precision, such as personal dose estimation, the combination of gradient-based methods and meta-heuristic optimization techniques provides more reliable results. This approach provides an important basis for the development of individualized decision support systems in the medical field.

### 3.2. Interpretation of Prediction Results Using XAI

In this section, the LIME (Local Interpretable Model-Agnostic Explanations) method was applied to evaluate the explainability of the regression-based machine learning model developed to calculate breast-specific radiation dose in thoracic CT examinations. The aim was to clearly identify which clinical or demographic characteristics influence the radiation dose prediction for individual patient-specific breast organ samples and to what extent they influence it.

**Figure 6:**
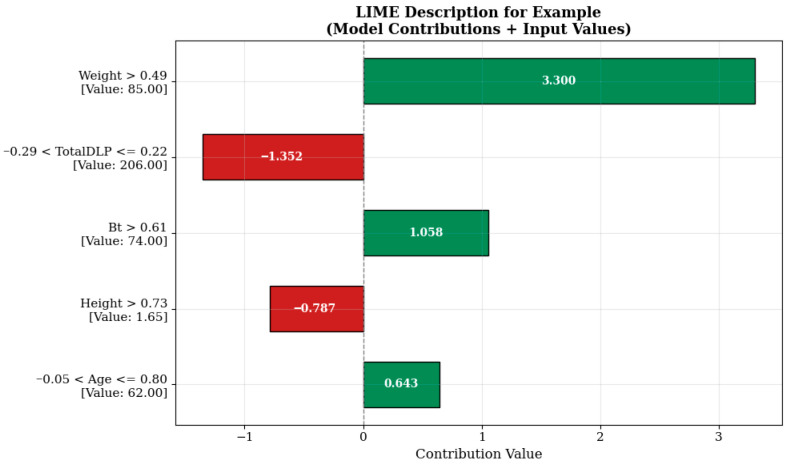
Prediction explanation for a sample patient.

When examining the LIME visualization in Figure 6, the model prediction for the relevant sample is significantly influenced by the weight variable represented by the condition “Weight > 0.49.” The value of this variable in the sample is 85.00, contributing a high positive value of +3.300 units to the model prediction. This indicates that the patient’s body weight is an important factor affecting imaging parameters in radiological examinations. It is known that increasing the radiation dose may be necessary to maintain imaging quality in individuals with higher body mass, and the model’s learning in this direction is consistent with clinical reality.

On the other hand, the TotalDLP variable (example value: 206.00), evaluated in the range ‘−0.29 < TotalDLP ≤ 0.22’, contributes negatively by reducing the estimate by −1.352 units. This effect may reflect the impact of dose history from previously administered imaging sessions or automatic dosimetric optimization systems. Similarly, the height variable, which is 1.65 m under the condition ‘Height > 0.73’, reduces the estimated value by −0.787 units, indicating that the model evaluates this anthropometric variable in a balancing manner. In contrast, the breast tissue quality (Bt) and age variables, expressed as ‘Bt > 0.61’ and ‘−0.05 < Age ≤ 0.80’, contribute +1.058 and +0.643 units to the model prediction, respectively. This situation indicates that factors such as breast density and age may be effective in determining radiological protocols and supports the model’s successful learning of this clinical information.

**Figure 7:**
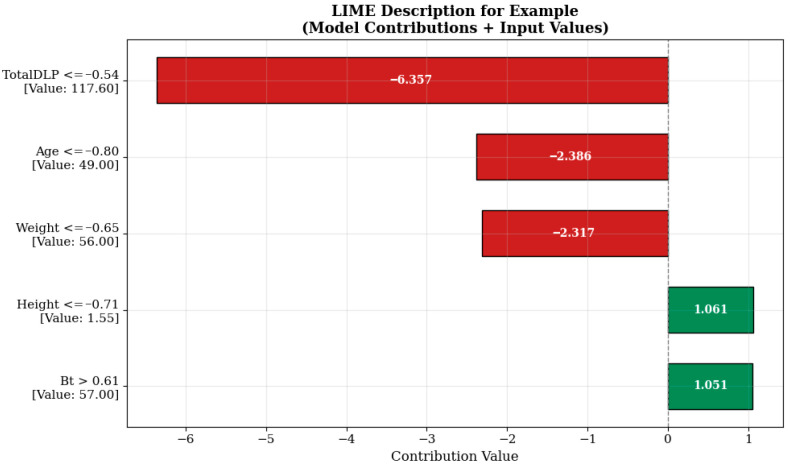
Prediction explanation for a sample patient.

The explanation in Figure 7 shows that the model’s prediction in this example is heavily influenced by negative contributions. In particular, the TotalDLP value, which is measured at 117.60 units in the example and is included in the condition ‘TotalDLP ≤ −0.54’, has been the variable that most strongly reduces the model prediction by −6.357 units. This indicates that the total radiation level predicted by the model may be limited in examples with lower initial doses. Similarly, the patient’s age (Age ≤ −0.80, 49.00 years) and body weight (Weight ≤ −0.65, 56.00 kg) are also among the other important factors that reduce the model prediction by −2.386 and −2.317 units, respectively. These variables reflect the impact of patient anthropometry on protocol selection in radiological applications. The model has successfully learned that radiation should be minimized in low-weight and younger patients, especially in sensitive areas such as the breast. In contrast, the height of 1.55 m under the condition ‘Height ≤ −0.71’ increased the model’s prediction by +1.061 units, and the breast tissue quality (Bt) of 57.00 under the condition ‘Bt > 0.61’ increased it by +1.051 units, acting as variables that positively contributed to the prediction. This finding suggests that there may be a possibility of dose increase due to imaging difficulties in shorter individuals and that breast structure characteristics are related to dosimetric requirements.

**Figure 8:**
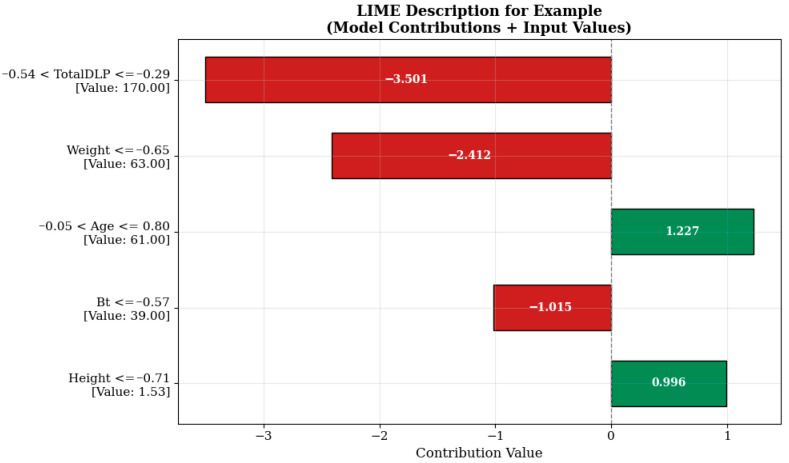
Prediction explanation for a sample patient.

The strongest negative contribution to the model estimate shown in Figure 8 comes from the Total DLP (total dose length multiplier) variable, which is measured at 170.00 units and falls within the range “−0.54 < TotalDLP ≤ −0.29” (−3.501 units). This indicates that the total dose level previously administered or recorded in the system has a reducing effect on the additional dose to be administered in a new scan. Such model behaviors are shaped in a manner consistent with clinical protocols aimed at limiting patients’ cumulative radiation exposure. Similarly, the body weight (‘Weight ≤ −0.65’, 63.00 kg) and breast tissue density (Bt ≤ −0.57, 39.00) variables also contributed negative effects of −2.412 and −1.015 units, respectively. This suggests that lower radiation doses may be sufficient, particularly in individuals with lower body mass and breast tissue with lower density. The age variable, evaluated under the condition “−0.05 < Age ≤ 0.80” and measured as 61.00 years, increased the model estimate by +1.227 units, while the height variable, measured as 1.53 m under the condition “Height ≤ −0.71”, increased the estimate by +0.996 units, thereby positively influencing the estimate. This situation may reflect the possibility of modifying protocol parameters to improve image quality in older and shorter patients.

## 4. Discussion

The frequency of CT examinations is increasing day by day. Radiation risk is a major concern, especially for radiosensitive organs such as the breast and lungs. Therefore, it is crucial to estimate the organ dose as accurately as possible in CT examinations and to determine dose optimization.

Numerous studies have evaluated organ doses in CT scans, and most of them have been performed using phantoms and MC simulation methods [14]. There are few studies that perform effective dose estimation with artificial intelligence support, and to our knowledge, there are no studies that model breast-specific organ doses with artificial intelligence. This makes our study valuable and debatable.

In a study by Wencheng Shao et al., organ doses in abdominal-pelvic CT examinations were estimated with ML. Reference organ doses were obtained using MC simulations on images. A support vector regression (SVR) model was trained based on radiomic features and reference organ doses and was used to estimate abdominal organ doses from CT scans [12]. In our study, model training was performed independently of images and MC. In models trained with MC simulation, the prediction accuracy of the model depends on the prediction accuracy of the MC simulation. Furthermore, image-based dose estimation is impractical. The MAPE range was 11.42–15.2% for chest organs. This rate is higher than that of the best model developed in our study (4.37%).

In a study by Matteo Ferrante et al., effective dose estimation was performed without the need for images using the ML and MC methods in CT scans. They developed three machines to learn algorithms. These algorithms performed better than effective dose estimation using k-factors. (MAE: 2.06; MAPE: 26%) [15]. In our study, five ML algorithms were developed using the MC method without relying on patient images. CatBoostPSO demonstrated superior performance across all metrics; it achieved the lowest MSE (0.3795), MAE (0.3846), and MAPE (4.37%) values while also having the highest R^2^ (0.9875) value. In our study, it was observed that breast-specific organ dose calculation showed higher performance.

An ML study calculating breast-specific organ dose was not found in our literature review. In another study using phantoms, the breast-specific organ dose calculated using the MC method in patients undergoing thoracic CT ranged from 6.5 to 28 mGy per examination, with an average breast organ dose of 15 mGy [14]. Due to the computational difficulty of the MC method used in the study and its lack of information about patient anatomy, it is difficult to apply in daily practice and has a high margin of error.

Our study developed a high-performance ML algorithm quickly using real patient data without relying on MC simulation. Algorithms trained using MC simulation are affected by the MC margin of error [16].

Although our study has the potential to provide high-performance breast-specific organ dose estimation, it still has many limitations and requires further research and detailed analysis. In the dose estimation model developed and tested in this study, the scans were performed with a single CT device at a single hospital. This resulted in limited data diversity. As no previous study has worked with an AI-supported model that calculates breast-specific organ dose, there is no study with which to compare our model. This study is preliminary and exploratory in nature. In the future, comparative analysis can be performed by adding more patients and CT devices.

Model performance was quantified using MAE and MAPE on the independent test set, and clinical acceptability was judged based on the magnitude and distribution of absolute and relative errors, in line with typical uncertainty ranges reported for organ dose estimation methods. No fixed institutional dose threshold was used; instead, the relatively low MAPE and narrow error distribution were interpreted as indicating that the prediction errors are unlikely to be clinically relevant.

Although TotalDLP is globally positively correlated with the predicted internal breast dose, the LIME explanations (Figure 6, Figure 7 and Figure 8) show that, in some intervals, TotalDLP can make a negative local contribution to the estimate. This behavior arises because the CatBoost model evaluates TotalDLP together with patient-specific variables (weight, BMI, breast thickness, age) and protocol history. In patients with lower body mass or younger age and/or previously high recorded TotalDLP, the model has learned a reduction in additional breast dose, consistent with protocol adjustments and automatic exposure-control mechanisms designed to limit cumulative radiation exposure.

## 5. Limitations

All scans were obtained on a single CT scanner using a standardized chest protocol; therefore, detailed acquisition parameters (pitch, rotation time, field of view) were not modeled as separate predictors, and their influence is captured only indirectly through the DLP and patient-related variables. Future multi-center studies including heterogeneous acquisition protocols are needed to quantify the specific impact of these parameters on breast dose.

In this study, breast size was modeled as a continuous mammographic breast thickness parameter in a cohort of real patients, and no discrete breast-size or phantom categories were defined. Consequently, model performance was not formally stratified by breast-size category, which should be explored in future multi-center and phantom-based studies.

One of the main limitations of this study is that all examinations were performed at a single institution, using a single CT scanner, and with a relatively homogeneous protocol. Consequently, the proposed breast dose estimation model has so far been validated only at a single center and on a single device. Therefore, future research will prepare for multicenter datasets involving different CT devices, vendors, and a wider range of acquisition parameters to externally validate the model and perform formal comparative analyses across systems and protocols.

## 6. Conclusions

This study proposes a machine learning-based approach that can predict the radiation dose received by breast tissue in thorax CT examinations with high accuracy using patient-specific parameters. Among the five different regression algorithms used in the study, the CatBoostPSO model performed the best by keeping the error rates at the lowest level (MSE: 0.3795, MAE: 0.3846, MAPE: 4.37%) and providing the highest coefficient of determination (R^2^: 0.9875). This finding suggests that gradient-based methods have a high potential for individualized dose estimation, especially when supported by meta-heuristic optimization techniques.

One of the most important contributions of the study is the ability to perform breast-specific dose estimation on real patient data without the need for Monte Carlo simulations or phantom-based methods. Thus, a fast, cost-effective and practical decision support tool that can be used in clinical practice has been developed. This enables personalized dose optimization and reduces radiation exposure to radiosensitive organs such as the breast. In addition, the evaluation of the model with explainable artificial intelligence (XAI) techniques transparently revealed which clinical and demographic variables were decisive in the prediction process and increased the adoptability of the method by clinicians.

## Figures and Tables

**Table 1:** Results of the experiments of all models.

Algorithms & Metrics	MSE	MAE	MAPE	R^2^	Hyperparameters
CatBoostPSO	0.3795	0.3846	0.0437	0.9875	‘iterations’: 201, ‘depth’: 4, ‘learning_rate’: 0.178688, ‘verbose’: 0, ‘random_state’: 42
GradientBoostPSO	1.1458	0.6010	0.0618	0.9622	‘learning_rate’: 0.05, ‘max_depth’: 3, ‘min_samples_leaf’: 1, ‘min_samples_split’: 5, ‘n_estimators’: 200
ExtraTreePSO	1.5067	0.7067	0.0761	0.9504	‘n_estimators’: 150, max_depth’: 12, ‘min_samples_split’: 5, ‘min_samples_leaf’: 2
AdaBoostPSO	2.4835	0.9006	0.0979	0.9182	‘n_estimators’: 97, ‘learning_rate’: 0.41181, ‘estimator’: DTRegressor (max_depth = 7, random_state = 42)
RandomForestPSO	2.5335	0.8658	0.0885	0.9165	‘n_estimators’: 50, ‘max_depth’: 9, ‘min_samples_split’: 2

## Data Availability

The data presented in this study are available on request from the corresponding author due to ethical approval limitations and patient confidentiality.

## Abbreviations

BMI	Body mass index
CT	Computed Tomography
DLP	Dose length product
IR	Internal Radiation
ML	Machine learning
MAE	Mean Absolute Error
MAPE	Mean Absolute Percentage Error
MSE	Mean Squared Error
mGy	Milligray
MC	Monte Carlo
PSO	Particle Swarm Optimization
ROI	Patient-specific dose estimation
ROI	Regions of interest
mA	Tube current
kV	Tube voltage
LIME	Local Interpretable Model-Agnostic Explanations
CTDIvol	Volumetric CT dose index
